# Analysis of chromatin fibers in Hela cells with electron tomography

**DOI:** 10.1007/s41048-015-0009-9

**Published:** 2015-08-07

**Authors:** Xiaomin Li, Hongli Feng, Jianguo Zhang, Lei Sun, Ping Zhu

**Affiliations:** National Laboratory of Biomacromolecules, Institute of Biophysics, Chinese Academy of Sciences, Beijing, 100101 China; Center for Biological Imaging, Institute of Biophysics, Chinese Academy of Sciences, Beijing, 100101 China; University of Chinese Academy of Sciences, Beijing, 100049 China

**Keywords:** Chromatin fiber, Hela cell, Electron tomography, Chemical fixation, High-pressure freezing, Cryo-ultramicrotomy, DualBeam-FIB

## Abstract

The presence and folding pattern of chromatin in eukaryotic cells remain elusive and controversial. In this study, we prepared ultra-thin sections of Hela cells with three different fixation and sectioning methods, i.e., chemical fixation, high pressure freezing with freeze substitution, and cryo-ultramicrotomy with SEM-FIB (focused ion beam), and analyzed in vivo architecture of chromatin fibers in Hela nuclei with electron tomography technology. The results suggest that the chromatin fibers in eukaryotic Hela cells are likely organized in an architecture with a diameter of about 30 nm.

## Introduction

The folding of chromatin in eukaryotic cells is closely related to the genetic transcription, replication and repair (Horn and Peterson 2002; Luger et al. 1997). Packaging of DNA in eukaryotic cells is hierarchical. The linear “beads-on-string” arrangement of nucleosomes, which is formed by histone octamers (H2A:H2B:H3:H4 = 2:2:2:2) (Luger et al. 1997) wrapped by DNA, is regarded as the first level arrangement of chromatin (Huynh et al. 2005). Although the nucleosome had been structurally characterized by X-ray crystallography at 1.9 Å (Davey et al. 2002), how polynucleosomes are folded into 30-nm chromatin fibers, which are typically regarded as the secondary structure of DNA, is inconclusive. Based on the early studies of chromatin in different cells using a variety of methods (Bednar et al. 1995; Daban 2011; Gerchman and Ramakrishnan 1987; Grigoryev and Woodcock 2012; Kruithof et al. 2009; Robinson and Rhodes 2006; Schalch et al. 2005; Simpson and Stafford 1983; Widom et al. 1985; William et al. 1986; William and Langmore 1991), researchers had proposed two major types of model for the secondary chromatin structure, i.e., “one-start” solenoid model and “two-start” zig-zag model (Finch and Klug 1976; Horowitz et al. 1994; Robinson et al. 2006). Recently, using cryo-electron microscopy single particle analysis, we reconstructed the 3D structure of in vitro reconstituted 30-nm chromatin fibers at 11 Å resolution and found that chromatin fibers with two different nucleosome repeat lengths (NRLs, 12 × 177 and 12 × 187 bp) present a left-handed double helix structure (Song et al. 2014), which represents a considerable advance on the structure characteristics of chromatin fibers. However, the existence of 30-nm chromatin fibers in the nuclei of eukaryotic cells is still remained to be elucidated in vivo (Eltsov et al. 2008).

Extensive studies have been made previously on the organization of native chromatin fibers, including those in starfish sperm nuclei (Giannasca et al. 1993; Horowitz et al. 1994), chicken erythrocyte nuclei (Langmore and Paulson 1983; Woodcock et al. 1984), *Thyone briareus* (sea cucumber) sperm, *Necturus maculosus* (mud-puppy) erythrocytes (Athey et al. 1990; William et al. 1986; Woodcock 1994), and in other cells (Davies et al. 1974; Derenzini et al. 2014; Eltsov et al. 2014; Everid et al. 1970; Fakan and van Driel 2007; Fussner et al. 2011; Konig et al. 2007; Matsuda et al. 2010). An electron tomography (ET) study showed that continuously variable zig-zag nucleosomal ribbons could be observed in chicken erythrocyte nuclei, both in the native form in situ and in the isolated form (Horowitz et al. 1994). Nevertheless, the samples used in that study were chemically fixed, dehydrated, embedded in resin, and stained by heavy metal. It was argued that the results could be attributed to the probable structure rearrangement and surrounding background staining artifacts (Eltsov et al. 2008). To visualize the close-to-native chromatin in vivo, techniques with a better preservation of the native status of the nuclei, i.e., high-pressure freezing, cryo-sectioning, and cryo-electron tomography, are necessary (Scheffer et al. 2011). However, even with a vitrified sectioning of cells and the contrast transfer function (CTF) correction on the electron microscopic images, it is difficult to visualize the high-order structure of 30-nm chromatin fibers in situ (Eltsov et al. 2008; McDowall et al. 1986).

In this study, we performed ET analysis to visualize the native chromatin arrangement in vivo, by taking three different sample preparation methods, i.e., ultrathin-sectioning with chemical fixation, ultrathin-sectioning with high pressure freezing and freeze substitution, and plunge-freezing with focused ion beam (FIB) cryo-sectioning. Among them, the ultrathin-sectioning with chemical fixation, embedding in resin, and chemical staining provides good contrast for electron microscopy imaging. Both high-pressure freezing and plunge-freezing can preserve the frozen-hydrated sample at cryo-temperatures without dehydration and keep the sample in a close-to-native state (Scheffer et al. 2011). The FIB method is a novel alternative to cryo-ultramicrotomy for thinning of frozen-hydrated biological specimens, which has brought a lot of attentions due to its peculiar advantages (Rigort et al. 2010). ET is a useful technology that has the ability to obtain 3D architectures of both homogeneous and heterogeneous samples (Scheffer et al. 2011). In particular, cryo-electron tomography has the ability to visualize the molecular assemblies in the unaltered frozen-hydrated state at reasonably high resolution. Here, we tried to explore the architecture of chromatin fibers in Hela cells in situ by combining all of these technologies. The results suggest that chromatins are likely present in the nuclei of Hela cells with an architecture of fibers with a diameter of about 30 nm.

## Results and discussion

### EM analysis of 30-nm chromatin fibers in Hela S3 cells and isolated nuclei

It is well recognized that the isolated chromatins from chicken erythrocyte nuclei present a fiberic form in width of ~30 nm (Scheffer et al. 2011). For the Hela S3 cells, the arrangement of 30-nm fibers had also been observed in the isolated chromatins (Langmore and Paulson 1983). Nevertheless, how the chromatin is organized in situ still needs to be elucidated (Eltsov et al. 2008; McDowall et al. 1986). Besides the in vitro assembled 30-nm chromatin fibers (Song et al. 2014), our study suggested that chromatin fibers isolated from Hela nuclei present a similar two-start double helix form (unpublished data). In this study, we tried to examine the chromatin fibers in Hela cells in situ to clarify if 30-nm chromatin fibers present in nuclei in vivo (Giannasca et al. 1993; Horowitz et al. 1994).

**Fig. 1:**
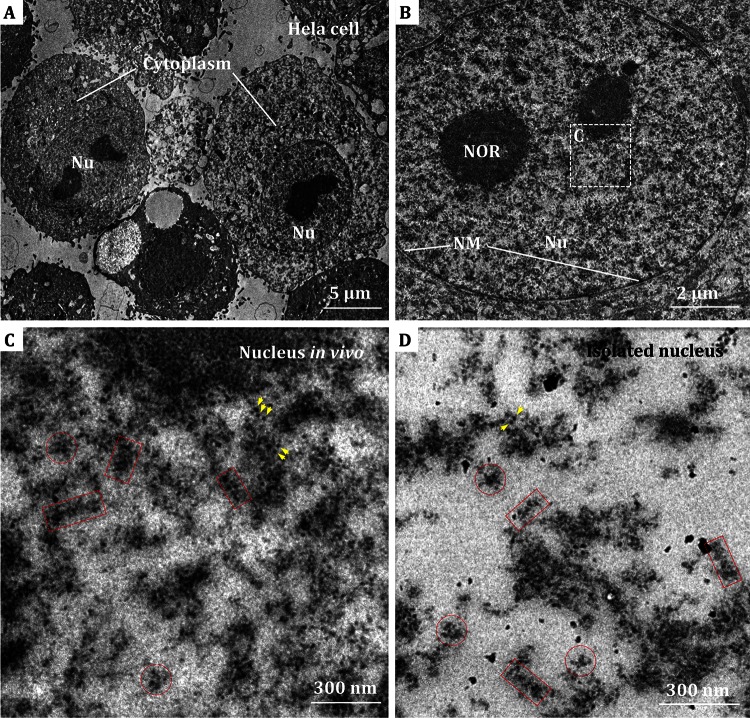
EM micrographs of conventional chemically fixed ultrathin sections of Hela cells and isolated nuclei. **A**, **B** Low magnification images of Hela cells. The nuclear membrane (NM) of nucleus (Nu) is indicated and the nucleolus (NOR) can be observed. **C** Enlarged view of the region highlighted in (**B**). The chromatin-like fibers with longitudinal section and cross section orientations are indicated by *red boxes* and *red circles*, respectively. The distinguishable nucleosome-like densities are indicated by *yellow arrowheads*. **D** EM image of isolated nuclei ultrathin section. The chromatin-like fibers and nucleosome-like densities are highlighted as in **C**

Firstly, we prepared the Hela S3 cell ultrathin-sections with conventional chemical fixation and heavy metal staining method, in order to get good contrast with electron microscopic imaging. To preserve the cell morphology, Hela cells were fixed in PBS buffer. Figure 1 shows the general appearance of the conventional ultrasection in 70 nm thickness of mitotic Hela S3 cells. The morphologies of the cells appear intact, and the periphery between nucleus and cytoplasm can be well defined in the low magnification microscopic image (Fig. 1A). As highlighted by the straight line in Fig. 1B, the double nuclear membrane is readily visible, which suggests that the structure of nucleus was preserved reasonably well during the chemical fixation process. In the EM images of the ultrathin-sectioned nuclei, compacted heterochromatin, and relatively incompact euchromatin are distinguishable (Fig. 1A, B). As shown in an enlarged view (Fig. 1C) of a region within the nucleus (white box indicated in Fig. 1B), a large amount of typical morphology of fibers, very likely attributed to the chromatins fibers, can be observed (red boxes in Fig. 1C). Overall, the chromatins in nuclei tend to form aggregates or clusters, which could be a part of the higher-order chromosome, although a part of chromatins appear loose and scattered. The chromatin fibers in various orientations, i.e., the cross sections (indicated by red circles in Fig. 1C) and longitudinally sections (red boxes in Fig. 1C), can be found in the ultrathin-sectioned nuclei. The longest chromatin fibers with longitudinally orientation were observed to extend as high as 300 nm. Furthermore, a number of heavily stained scattered dots with a size of ~11 nm, which are in the similar size range as that of individual nucleosomes, can be observed (yellow arrows in Fig. 1C). These observations suggest that the native chromatins are arranged in a diversified and heterogeneous manner in vivo.

We then isolated Hela nuclei from the cells following the previously reported protocols (Athey et al. 1990; Giannasca et al. 1993; William and Langmore 1991; Woodcock et al. 1984), and performed a similar chemical fixation, ultrathin-sectioning, and electron microscopic imaging analysis. The ultrathin sections of isolated Hela nuclei displayed distinctly separated fibers, most likely attributed to the chromatin fibers, as shown in representative EM images (Fig. 1D). Compared with those in the ultrathin-sections of the whole Hela cell, the chromatin fibers in isolated nuclei present similar architectures as in the cell. Different orientations of the chromatin fibers, e.g., cross sections and longitudinally sections, can also be visualized. It is worth noting that the ultrathin sections of isolated nuclei present a much cleaner background than that of nuclei in the cell. The contrast and signal-to-noise ratio of EM images of isolated nuclei are also better than that of whole cell. The chromatin-like fibers appear more apparent in the isolated nuclei (Fig. 1D), which was previously noticed in the study of chromatin fibers in starfish sperm (Horowitz et al. 1994). The 11 nm nucleosome-like dots can be recognized in the isolated nuclei sections. Unlike the chromatin in starfish sperm in which the chromatin fibers appear with sharp turns and folds (Horowitz et al. 1994), most of the fibers in the sectioned Hela nuclei appear with the longitudinal orientation. However, it is hard to track the fibers’ tendency in the EM images of Hela S3 cells, possibly due to the high variability and aggregation of chromatins. The diversity, flexibility and aggregation of native chromatin (Woodcock et al. 1984) and the hierarchical folding of chromatin at different cell cycle stages make it difficult to recognize the 30-nm fibers in the electron microscopic images. Our results suggest that an optimization on the cell cycle and sample preparations are necessary for the chromatin fibers characterization in vivo.

### Electron tomography reconstruction of isolated nuclei with high-pressure freezing and freeze substitution

**Fig. 2:**
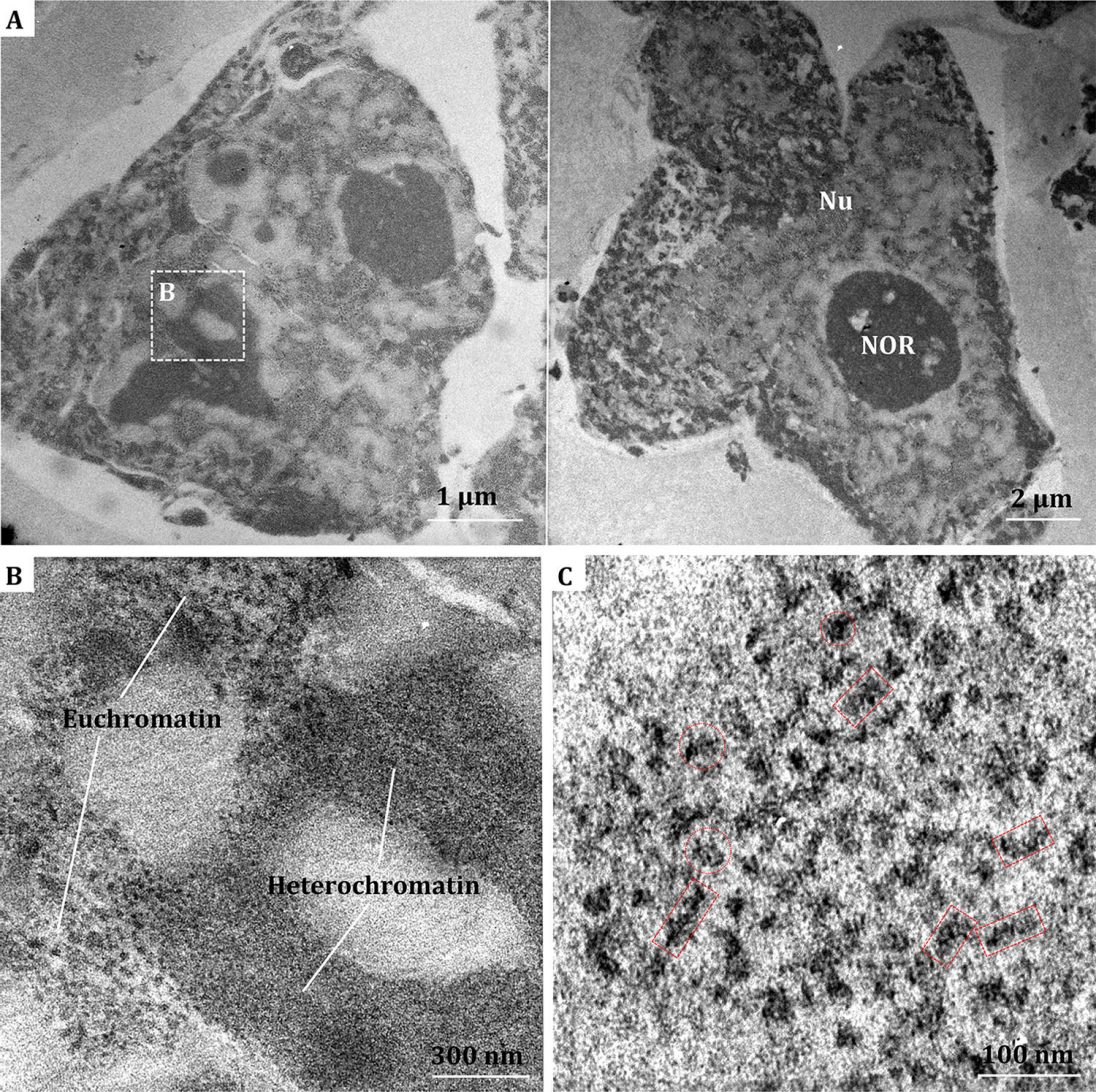
EM micrographs of isolated Hela nuclei with high-pressure freezing and freeze substitution. **A** Low magnification image of sectioned nuclei. **B** Enlarged view of the region highlighted in **A**. Presumptive euchromatin and heterochromatin are indicated. **C** Chromatin-like fibers with longitudinal-section and cross-section orientations are indicated by *red boxes* and *red circles*, respectively

As shown in the electron microscopic analysis discussed previously, the chromatin-like fibers likely present in the nucleus of Hela S3 cells, but the limited resolution of chemically fixed ultrathin section (Eltsov et al. 2008) made the pattern recognition of the nucleosomes in mitotic Hela S3 cells difficult. In order to analyze the three-dimensional structure of native chromatin in situ with reasonably high resolution, we tried to fix the isolated nuclei with a high-pressure freezing and freeze substitution process. The frozen-hydrated sample was then embedded at room temperature after freeze substitution. This procedure can preserve the chromatin fibers in a better way than that of the conventional chemical fixation method described above as all the macromolecules and supramolecules in the nucleus were immobilized at the close-to-native state (Eltsov et al. 2008). The embedding process prevents the sample from ice crystal contamination and the staining of ultrathin sections provides high contrast, which could facilitate the visualization of native chromatin fibers in situ. Figure 2 displays the EM images of isolated Hela nuclei with high pressure frozen fixation. The boundary of the nucleus appears intact (Fig. 2A), and more structural details could be identified than that of the chemical-fixed sample (Fig. 2B, C). The heterochromatin regions (indicated in Fig. 2B) appear with compacted chromatin agglomeration, which is frequently observed in the Hela nuclei. In the region with euchromatin, chromatin fiber filaments with both longitudinally sections and cross sections orientations can be observed (Fig. 2B, C).

**Fig. 3:**
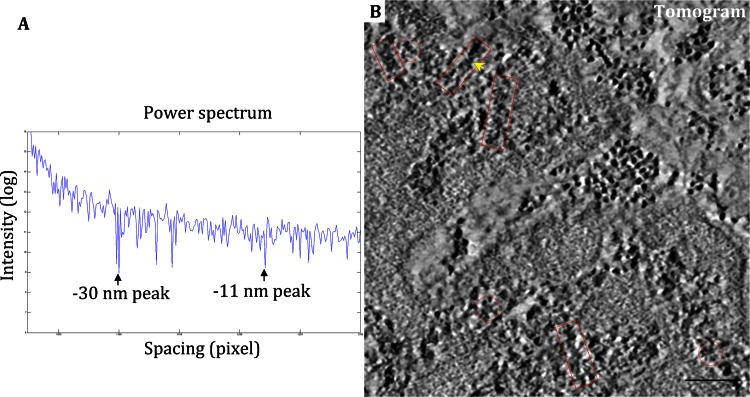
Characterization of chromatin fibers in the isolated Hela nuclei with high-pressure fixation. **A** The power spectrum diagram of the ultrathin-sectioned nuclei image shows peaks at ~11 and ~30 nm, respectively. **B** A tomogram slice of the ultrathin-sections after electron tomographic reconstruction. The chromatin-like fibers with longitudinal-section and cross-section orientations are indicated by *red boxes* and *red circles*, respectively. The fiber with two rows and twisting position is highlighted by *yellow arrow*. (*Scale bar* 100 nm)

The power spectrum analysis of EM images was previously used to inspect the existence of 30-nm chromatin fibers in Hela cells (Eltsov et al. 2008) and in isolated chicken erythrocyte nuclei (Scheffer et al. 2011). We performed a similar analysis on the EM images of isolated Hela nuclei after high pressure freezing and freeze substitution. Peaks at ~11 and ~30 nm positions appear distinguished in the power spectrum diagram (Fig. 3A), which implies that the chromatin in Hela cells could be folded into regular 30-nm high-order fibers with the 11-nm nucleosome as the elementary unit (Langmore and Paulson 1983).

To further verify the presence of 30-nm fibers, we made an ET analysis on the ultrathin sections of isolated Hela nuclei with high pressure freezing and freeze substitution. ET data were collected at the positions with separated fibers visible. The collection of tilt series data of the plastic ultrathin section was initially unsuccessful, because the shrinkage and the associated electron irradiation of plastic sections made it difficult to track the images well at different tilt angles. At the same time, the serious charging effect of the plastic sections produced a large drift on the acquired images. To resolve these issues, we coated a thin layer of carbon film on each side of the EM grid on top of the sections. The targeted regions were then irradiated by electron beam for several minutes. These procedures seem to be helpful for the conductivity improvement and drift prevention, and the tilt series had been successfully acquired.

In the electron tomograms, two orientations of chromatin fibers in width of ~30 nm are readily visible (Fig. 3B, indicated by red boxes and circles in the snapshots of tomographic slice). Although the flexibility and diversity of the chromatin fibers make it impossible to perform a sub-volume averaging, a large amount of individual intense electron densities, likely attributed to the nucleosomes, are distinguishable (Fig. 3B). The electron tomograms show that the chromatin fibers present a pattern of two rows with a twisting conformation (highlighted by yellow arrow in Fig. 3B). These results suggest that a “two start” architecture of chromatin fibers with a diameter of about 30 nm likely presents in Hela nuclei.

### Large-scale analysis of Hela nuclei with SEM–FIB and cryo-electron tomography

In addition to high-pressure freezing, fast freezing is also one of the common methods to prepare the frozen specimens. However, the diameter of Hela nuclei is as large as 7–10 μm and the electron beam cannot penetrate the vitrified sample with such a thickness. Cryo-ultramicrotomy sectioning of vitrified materials is a conventional method to acquire the sample for electron microscopic study, but this method inevitably suffers from distortions and deformations caused by the mechanical cutting process, and forms unavoidable sample compressions in the cutting direction, which introduce significant artifacts to the later ET analysis. In contrast to mechanical sectioning, cryo-FIB milling can be applied to the frozen-hydrated material to thin the native samples and make them transparent enough (50–200 nm) for TEM imaging (Rogort et al. 2012), without introducing heat-induced devitrification effect.

To reveal the characteristics and architecture of chromatin in situ, we applied state-of-the-art cryo-FIB thin sectioning and cryo-electron tomography technologies on the three-D reconstruction of Hela cells. The EM grids made of molybdenum were chosen due to their good characteristics of hardness, compared with copper and gold grids. The Hela cells in optimized concentration were fast frozen in a cryo-plunger, and transferred to a dual beam SEM to be thinned with FIB milling. The cryo-FIB thinned samples on EM grids were then subjected to ET analysis.

**Fig. 4:**
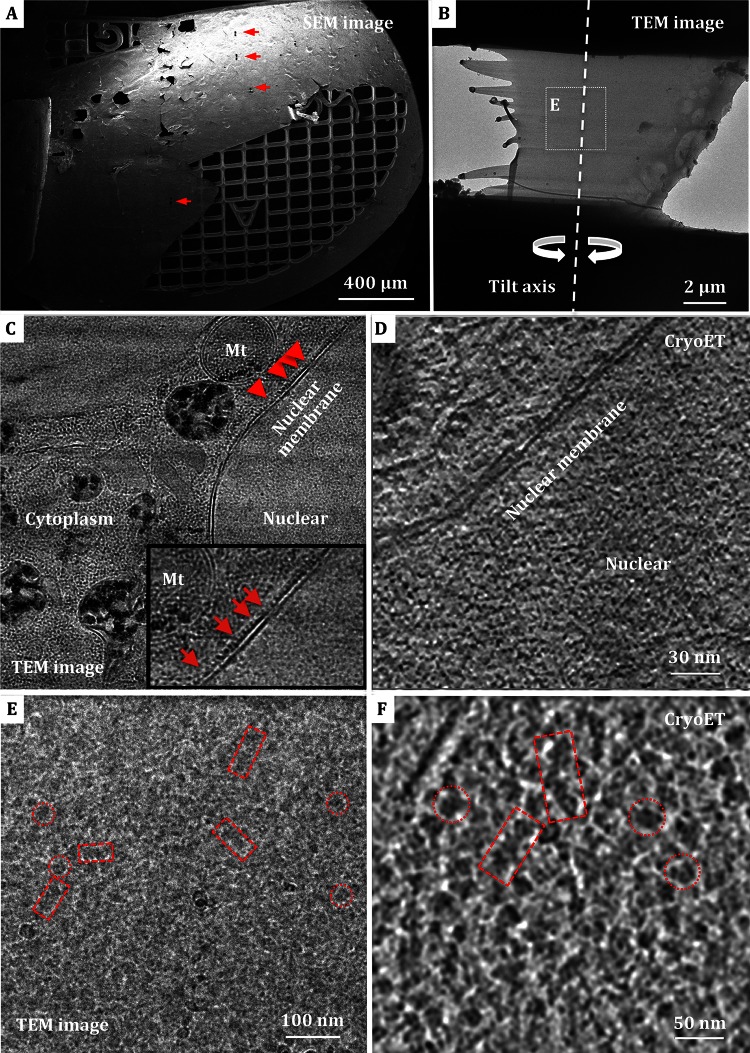
Cryo-ultramicrotomy analysis of Hela cells and isolated nuclei. **A** SEM micrograph of frozen-hydrated Hela cells embedded in ice and attached to Mo-grid with a thin layer of holey carbon supporting film. The lamellas yielded by FIB milling are indicated by *red arrows*. **B** Low magnification EM micrograph of the lamella of isolated nuclei. The *tilt* axis of cryo-electron tomography (cryo-ET) data collection is marked with a *dashed line*. **C** TEM image of cryo-FIB milled Hela cells lamella, which shows distinguishable mitochondria (Mt), double nuclear membrane and cytoplasm. The *inset* image displays the presumptive ribosomes attached to nuclear membrane. **D** A tomographic slice of cryo-lamella of Hela cells after reconstruction. **E** Enlarged view of the region *highlighted* in **B** with a low dose exposure. **F** A tomographic slice of the nuclei cryo-lamella after reconstruction. Chromatin-like fibers with longitudinal-section and cross-section orientations are indicated by *boxes* and *circles*, respectively, in both **E** and **F**

The cells with protruding morphology appearance in SEM are versatile enough to be selected as the milling targets (Rigort et al. 2010). Samples at four or five positions located at the center of the EM grid (indicated by red arrows in Fig. 4A) were selected and thinned. The positions of cryo-FIB thinning were marked in SEM with the coordinates on the EM grid and were looked up at a low magnification after the grid was transferred to TEM for cryo-electron tomography analysis. Figure 4B displays a representative area of the cryo-sections with a length of ~12 μm and a width of ~7 μm. Little ice crystal contamination was observed in this area (Fig. 4B), suggesting a reasonably well-done cryo-sectioning and grid transferring process. In the TEM image (Fig. 4C), the cytoplasm, mitochondria, and double nuclear membrane are distinguishable. We then performed cryo-ET analysis on the nuclei regions to characterize the architectures of chromatin fibers in the close-to-native status in situ. Tilt series were collected along a single tilt axis (Fig. 4B) with attentions paid to avoid the electron irradiation damage. After reconstruction, no regular high-order structure is clearly visible, except for the filamentous materials (Fig. 4D).

In order to enhance the probability of milled nuclei region, we then used isolated nuclei to do cryo-FIB sectioning and cryoET reconstruction. Recognizable fiber densities, most likely of the chromatin fibers, with the width of ~30 nm are readily visible with good contrast (Fig. 4E, red boxes and circles) in the TEM image. The reconstructed tomograms also display fiber densities with width in the range of 30 nm, suggesting the presence of 30-nm chromatin fibers in Hela nuclei (Fig. 4F). The chromatin fibers in the cryo-tomograms of Hela nuclei in situ appear similar to those observed in the chemically fixed and high-pressure frozen Hela nuclei, and in accordance with the conventional mechanical cryo-sections of isolated chicken erythrocyte nuclei (Scheffer et al. 2011). These results provide further evidence to the presence of 30-nm high-order chromatin fibers in vivo and shed lights on the three-dimensional structure of chromatin fibers in situ. Although the resolution of the cryo-tomograms is not high enough for us to track the path of DNA and to identify individual nucleosomes, our study suggests that it is technically feasible for a large-scale observation of isolated Hela nuclei with SEM–FIB and cryo-electron tomography.

### Conclusion

We have examined in situ architecture of chromatin fibers in eukaryotic Hela cells with ET analysis based on three different sample preparing technologies, i.e., conventional chemical fixation, high-pressure freezing and freeze substitution, and fast frozen and cryo-FIB sectioning. Although the sample preparation methods preserved the native structures to variant extents, chromatin fibers with width of ~30 nm have been observed in all of the ultrathin-sections prepared by these different methods. ET analysis on both high-pressure frozen-substituted sections and cryo-FIB sections of Hela nuclei, which preserve the chromatin fibers at the most close-to-native state in vivo, suggests that the chromatin fibers may present in Hela cells with a diameter of about 30 nm. Further studies and more efforts to improve the ET resolution, which is high enough to track the path of DNA and recognize individual nucleosomes, are critical to resolve the folding pattern of native chromatin fibers in vivo.

## Materials and methods

### Culture of Hela cells and isolation of Hela nuclei

The Hela cells were cultured in medium of DMEM with 10% FBS and 1% (100×) penicillin–streptomycin solution, and were passaged by 0.25% trypsin-EDTA for 2 min. A cell scraper was used to collect the cells, which were used for ultrathin-sectioning later. For the nuclei isolation, the suspension solution was centrifugated at 3000×*g* for 5 min and the supernatant was discarded. The cells were then suspended and centrifuged in PBS (with 0.5 g/L Mg^2+^) for three times. After that, the cells were resuspended in Buffer A (0.25 mol/L sucrose, 60 mmol/L KCl, 15 mmol/L NaCl, 10 mmol/L MES, 5 mmol/L MgCl_2_, 1 mmol/L CaCl_2_, 0.5% Triton X-100, 0.1 mmol/L PMSF, 0.5 mmol/L DTT, 0.5 mmol/L sodium metabisulfite, 0.5 mmol/L benzamidine-HCl, pH 6.5) for 5 min, and grounded until the suspension presented a milk-white color. The sediment was retained after centrifugation for 10 min at 5000×*g*. The nuclei were finally collected after grinding and centrifugation for two times.

### Ultrathin-sectioning of Hela cells and nuclei with chemical fixation

The cells collected by the scraper and the isolated Hela nuclei were pre-fixed by adding 100 μL 2.5% glutaraldehyde for 2.5 h at 4 °C and post-fixed by adding 100 μL 2% osmium trioxide for 1 h at 4 °C. The dehydration was proceeded step-by-step via adding 30, 50, 70, 90, 95, and 100% ethanol, respectively. Propylene oxide (PO) and resin were added to complete replacement and osmosis step-by-step with different ratios. The samples were embedded and polymerized in resin for 12 h at 35 °C, 12 h at 45 °C and 24 h at 60 °C successively. The embedded block was cut into ultrathin sections with about 70 nm thickness and transferred to a formvar film covered copper grid, and stained with 4% uranyl acetate and lead citrate.

### Ultrathin-sections of Hela nuclei by high-pressure freezing and freeze substitution

The isolated Hela nuclei with the cryo-protectant of hexadecane were frozen by a high pressure freezer (Leica EM PACT2). Freeze substitution was performed in a substitution unit (Leica EM AFS2) in dry acetone with 2% osmium tetroxide at −90 °C for 24 h, and then gradually warmed up to −20 °C for 8 h and 0 °C for 2 h. After being washed with dry acetone at 0 °C for 30 min, the samples were warmed up to room temperature and infiltrated with acetone and resin for several hours. The samples were then embedded into resin in embedding molds and put in a 60 °C oven for 24 h, and finally sectioned with microtome (Leica EM UC6) with approximate 70 nm thickness. The ultrathin sections were then stained by 4% uranyl acetate and lead citrate.

### Fast freezing of Hela nuclei and cryo-ultrasection with SEM–FIB

To acquire the cryo-samples embedded in the unaltered frozen-hydrated state, we used FEI Vitrobot to freeze the Hela nuclei. PBS was added into the isolated nuclei and about 3.5 μL sample was absorbed onto a molybdenum grid and dropped into liquid ethane for rapid freezing. The fast frozen nucleuses on Mo-grid were transferred into a dual beam-SEM (FEI Helios Nanolab 600i) with a specially designed clamping and transferring apparatus and thinned with FIB in the SEM. The vitrified samples were imaged in SEM at 10–30 kV acceleration voltages to inspect the thickness of the vitrified ice. The nuclei in regions with relatively thin ice, typically 4–5 areas within the center of the mesh, were selected for FIB milling. The accelerate voltage of FIB was set to 30 kV, and the beam current was set to 0.79 nA for rough milling, which was then changed to 0.80 pA for the fine milling. The FIB was adjusted in an angle of 10°–15° with specimen. The work distance of FIB was kept at 4 mm and the stage temperature was kept under −180 °C. The thin-sectioned nuclei samples on the Mo-grids were stored in a liquid nitrogen container for future ET analysis.

### Imaging of TEM, data collection and processing of electron tomography

The imaging of the conventional chemically fixed ultra-sectioned samples was done in the FEI Tecnai Spirit TEM (120 kV). The imaging and ET data collection of ultrathin-sectioned nuclei after high-pressure freezing, freeze substitution, and fixation were also done in the FEI Tecnai Spirit. To reduce the electron charging effects, both sides of the EM grids were coated with a thin layer of carbon film (2–5 nm in thickness). Single-axis tilt series were collected at a 1.5° increment between −60° and +60° in the FEI Tecnai Spirit equipped with an FEI Eagle CCD camera at 2 k × 2 k pixels, using FEI Xplore 3D software package. The acceleration voltage was 100 kV and the magnification was 23,000× with the pixel size of 1.02 nm/pixel. The dose for each tilted image was about 1 e/Å^2^, and the defocus was set to 3 μm. In total, 25 sets of tomographic data were collected. They were then aligned and reconstructed with PROTOMO software package. Median filtering was applied to the reconstructed tomograms to enhance the signal-to-noise ratio. MATLAB was used for the power spectrum analysis.

The imaging and cryo-electron tomography data collection of cryo-ultrasectioned samples with SEM–FIB were done in a 200 kV TEM with FEG (FEI Talos F200C). The automatic software package of FEI Tomography was used for the tilt series data collection using an FEI Ceta camera at 2 k × 2 k pixels. The magnification was 36,000× with a pixel size of 0.58 nm/pixel. The defocus was set to 6 μm and the dose of each tilted frame was about 1 e/Å^2^. Single-axis tilt series were collected at 1.5° increment. Seven sets of tomographic data were acquired, and then aligned and reconstructed in PROTOMO software package. Median filtering and non-linear anisotropic diffusion filtering distributed in IMOD software package were applied to the tomograms to enhance the contrast and signal-to-noise ratio.
